# Distinct Slow-Wave Activity Patterns in Resting-State Electroencephalography and Their Relation to Language Functioning in Low-Grade Glioma and Meningioma Patients

**DOI:** 10.3389/fnhum.2022.748128

**Published:** 2022-03-24

**Authors:** Nienke Wolthuis, Ingeborg Bosma, Roelien Bastiaanse, Perumpillichira J. Cherian, Marion Smits, Wencke Veenstra, Michiel Wagemakers, Arnaud Vincent, Djaina Satoer

**Affiliations:** ^1^Center for Language and Cognition Groningen (CLCG), University of Groningen, Groningen, Netherlands; ^2^Department of Neurology, University Medical Center Groningen, Groningen, Netherlands; ^3^National Research University Higher School of Economics, Moscow, Russia; ^4^Department of Neurology, University Medical Center Rotterdam, Rotterdam, Netherlands; ^5^Division of Neurology, Department of Medicine, McMaster University and Hamilton Health Sciences, Hamilton, ON, Canada; ^6^Department of Radiology & Nuclear Medicine, Erasmus MC - University Medical Center Rotterdam, Rotterdam, Netherlands; ^7^Brain Tumour Centre, Erasmus MC Cancer Institute, Rotterdam, Netherlands; ^8^Department of Rehabilitation Medicine, University Medical Center Groningen, University of Groningen, Groningen, Netherlands; ^9^Department of Neurosurgery, University Medical Center Groningen, Groningen, Netherlands; ^10^Department of Neurosurgery, Erasmus MC – University Medical Center Rotterdam, Rotterdam, Netherlands

**Keywords:** brain tumour, glioma, meningioma, language, slow-wave brain activity, EEG

## Abstract

**Introduction:**

Brain tumours frequently cause language impairments and are also likely to co-occur with localised abnormal slow-wave brain activity. However, it is unclear whether this applies specifically to low-grade brain tumours. We investigate slow-wave activity in resting-state electroencephalography (EEG) in low-grade glioma and meningioma patients, and its relation to pre- and postoperative language functioning.

**Method:**

Patients with a glioma (*N* = 15) infiltrating the language-dominant hemisphere and patients with a meningioma (*N* = 10) with mass effect on this hemisphere underwent extensive language testing before and 1 year after surgery. EEG was registered preoperatively, postoperatively (glioma patients only), and once in healthy individuals. Slow-wave activity in delta- and theta- frequency bands was evaluated visually and quantitatively by spectral power at three levels over the scalp: the whole brain, the affected hemisphere, and the affected region.

**Results:**

Glioma patients had increased delta activity (affected area) and increased theta activity (all levels) before and after surgery. In these patients, increased preoperative theta activity was related to the presence of language impairment, especially to poor word retrieval and grammatical performance. Preoperative slow-wave activity was also related to postoperative language outcomes. Meningioma patients showed no significant increase in EEG slow-wave activity compared to healthy individuals, but they presented with word retrieval, grammatical, and writing problems preoperatively, as well as with writing impairments postoperatively.

**Discussion:**

Although the brain-tumour pathology in low-grade gliomas and meningiomas has a different effect on resting-state brain activity, patients with low-grade gliomas and meningiomas both suffer from language impairments. Increased theta activity in glioma patients can be considered as a language-impairment marker, with prognostic value for language outcome after surgery.

## Introduction

Approximately 10 per 100,000 individuals are diagnosed with a primary brain tumour every year (De Robles et al., 2015). When located in the left hemisphere, it is estimated that nearly half of these tumours cause language impairments (Davie et al., 2009). So far, much research has been dedicated to language functions in glioma patients (Papagno et al., 2012; Satoer et al., 2016; Antonsson et al., 2018), however it is unclear whether language deficits also occur in patients harbouring meningiomas. Low-grade gliomas grow relatively slowly, but typically infiltrate neural tissue important for sensorimotor and language functions causing preoperative impaired language performance in glioma patients (Van Kessel et al., 2017). When comparing abilities before surgery to those 3–6 months after surgery, language functioning in glioma patients may not change or may decline, as systematically reviewed by Satoer et al. (2016). However, it is possible that the applied aphasia tests, traditionally designed for stroke population, are not sufficiently sensitive to detect the mild language problems in low-grade glioma patients (Satoer et al., 2018). In contrast to gliomas, meningiomas grow from the meninges, thus do not infiltrate functional areas, but rather compress and distort the surrounding brain tissue (Wei et al., 2007). Furthermore, in meningioma patients, subcortical white matter pathways may be displaced, but do stay intact (Campanella et al., 2014). For these reasons, one may expect little or no impairments in cognitive functioning. However, a systematic review by Meskal et al. (2016) mentions that the majority of meningioma patients suffer from impairment of one or more cognitive functions (e.g., memory, attention, and executive functioning) before surgery. These functions tend to mostly improve after surgery (Meskal et al., 2015). Little is known about language functioning in meningioma patients.

Language impairments have an immense impact on everyday domestic, social, and professional activities, and, thus, on the quality of life (Hilari et al., 2012). These impairments can affect different language modalities (speech production, comprehension, reading, and writing) and linguistic levels, such as phonology (speech sounds), semantics (meaning), and grammar (word and sentence structure). Adequate language functioning relies on a large-scale, usually left-lateralised neural network, involving a large part of the perisylvian cortex and the underlying white-matter pathways (Duffau, 2014). Injury to these subcortical tracts increases the risk of language decline and permanent impairments after brain-tumour surgery (Bello et al., 2007; Trinh et al., 2013).

Brain injury, principally white-matter injury, is known to co-occur with increased slow-wave activity: an excess of focal brain activity in the delta (0.5–4 Hz) and theta (4–8 Hz) frequency bands (Lüders and Noachtar, 2000). Increased slow-wave activity is considered pathological, indicative of brain dysfunction, when present in adults during wakeful, resting conditions (Lüders and Noachtar, 2000). Slow-waves are presumed to be induced by injured tissue around the lesion, specifically by white-matter injury, and generated by cortical areas overlying the lesion (Ball et al., 1977; Gloor et al., 1977). Increased slow-wave activity has been observed in brain-tumour patients (Baayen et al., 2003; De Jongh et al., 2003; Oshino et al., 2007). However, these studies concerned heterogeneous groups of brain-tumour patients, including different tumour types and grades. It is unclear whether increased slow-wave activity is evident in low-grade glioma and meningioma patients, in whom the tumour has developed over a long time.

Gliomas are intra-axial, infiltrative tumours, representing the most frequently occurring primary brain tumours, of which 20% are low-grade (World Health Organisation [WHO] grade I, II) (Ho et al., 2014; Louis et al., 2016). One-third of primary brain tumours concern meningiomas; these are extra-axial, non-infiltrative tumours, of which 95% are low-grade (WHO grade I) (Wiemels et al., 2010). Low-grade gliomas and meningiomas have different tumour characteristics with different effects on peritumoural brain tissue (infiltration vs. compression respectively), possibly affecting slow-wave activity patterns differently. Patients with a low-grade brain tumour are usually young individuals with a relatively long life-expectancy after surgery (Van Alkemade et al., 2012; Ho et al., 2014). Preservation of language functioning is therefore crucial for these patients to successfully carry out daily activities and return to work.

The relationship between EEG slow-wave activity and language functioning in brain tumour patients has not been studied yet. For cognitive functions, it has been shown that increased slow-wave activity in low-grade glioma patients is associated with poorer working memory, information processing, and executive functioning (Bosma et al., 2008). Also, a larger area of increased delta activity was related to poorer functional outcomes after brain-tumour surgery (Oshino et al., 2007).

In patients with non-tumour-related brain injury, for example, due to stroke or neurodegenerative disease, slow-wave activity has been associated with language-impairment severity (Jabbari et al., 1979; Chapman et al., 1989; Finitzo et al., 1991; Claus et al., 2000; Szelies et al., 2002; Hensel et al., 2004; Meinzer et al., 2004; Van der Hiele et al., 2007; Olde Dubbelink et al., 2013; Kielar et al., 2019). Moreover, slow-wave activity has been used to predict the course of language recovery in post-stroke aphasia. Little or no slow-wave activity was indicative of better language recovery, whereas a high level of slow-wave activity was indicative of poor language recovery (Tikofsky et al., 1960; Jabbari et al., 1979; Szelies et al., 2002; Hensel et al., 2004).

The current study investigates whether slow-wave activity has clinical relevance for the course of language functioning in patients undergoing low-grade glioma or meningioma surgery. We use electroencephalography (EEG) for brain-activity registration because it is relatively inexpensive and commonly used in clinical practice. Hence, potential novel findings are clinically applicable and may aid in perisurgical patient care, such as treatment planning, counselling, and language rehabilitation. Additionally, we attempt to gain more insight into the underlying neural mechanisms of language impairments, for which the main language modalities and linguistic levels (combined into “language domains”) are taken into account. Our research questions are:

1) Do low-grade glioma and meningioma patients suffer from language impairments?
2) Do low-grade glioma and meningioma patients have more slow-wave activity than healthy individuals in their EEG preoperatively and 1 year postoperatively?
3) And if so, is this increase related to preoperative language functioning and 1 year postoperative language outcome?

## Materials and Methods

### Participants

Patients with a presumed low-grade glioma (*N* = 15) and patients with a presumed grade I meningioma (*N* = 10), who were scheduled to undergo surgery at the University Medical Centre Groningen (UMCG) or the Erasmus MC University Medical Centre Rotterdam (Erasmus MC), were invited to participate in the study.

Inclusion criteria were:

• Intracranial, supratentorial, untreated tumour infiltrating into or pressing on the language-dominant hemisphere. This was evidenced by functional MRI in 9/15 glioma patients, if language lateralisation was unknown: right-handed patients with a left-sided tumour (as left hemispheric language lateralisation is found in the majority of right-handed people; Benson et al., 1999) were included;
• Tumour diameter >3 cm (meningiomas only);
• Aged between 18 and 75 years;
• In case of epilepsy, seizures under control with anticonvulsants.

Exclusion criteria were:

• Non-native Dutch speaker;
• History of a medical, neurological or psychiatric condition known to affect language or cognitive functioning;
• History of substance abuse;
• Consistent use of dexamethasone preoperatively;
• Previous brain surgery or cranial radiation therapy.

Glioma patients underwent tumour resection with an awake procedure and meningioma patients underwent surgery under general anaesthesia. For both aetiologies, a control group was composed of healthy volunteers (*N* = 15 matched to gliomas, *N* = 9 matched to meningiomas), matched for age, education, and gender (often proxies of the patients). The same exclusion criteria applied for these groups. This multicentre study was approved by the medical, ethical review board of the UMCG, which was also valid to the Erasmus MC. All participants gave written informed consent.

### Procedure

Both patient groups underwent extensive language assessment at two time points: T1 = before surgery (glioma patients: mean 33 days, range 6–83; meningioma patients: mean 20 days, range 1–48); and T2 = 12 months after surgery (glioma patients: mean 12.61, range 11.05–15.22; meningioma patients: mean 11.97, range 11.21–12.49). EEG registration was performed at T1 and at T2 in glioma patients, at T1 in meningioma patients^1^, and once in healthy participants.

### Language Assessment

A selection of standardised language tests was conducted, assessing a wide range of linguistic abilities. Tests included object naming (De Witte et al., 2015), action naming in sentence context (Olde Dubbelink et al., 2013), category fluency (Szelies et al., 2002), letter fluency (Schmand et al., 2008), reading and writing (Van Ierschot, 2018), and subtests of the Diagnostic Instrument for Mild Aphasia (DIMA) (Satoer et al., 2019): repetition, semantic odd-picture-out, and sentence completion.

Raw scores were normalised by conversion to z-scores, using pre-existing normative data from a healthy population. The tests were divided over six language domains (Table 1) to cover performance in three language modalities (speech production, reading, and writing) and at three linguistic levels (phonology, semantics, and grammar). For each patient, the domain score equalled the average z-score of the tests in that domain. Domain scores below -1.5 were considered to reflect an impairment.

**TABLE 1:** Language domains under investigation with their corresponding tests.

Domains	Tests	Description	Normative data (N)
Word retrieval	Object naming (De Witte et al., 2015)	Naming objects depicted on black-and-white drawings with the introduction *This is*…	145
Phonology	Repetition (Satoer et al., 2019)	Repetition of three-syllable words, compound words, non-words, and sentences.	103
	Letter fluency (Schmand et al., 2008)	Producing words starting with a given letter within 60 sec.	570
Semantics	Semantic odd-picture-out (Satoer et al., 2019)	Out of three black-and-white drawings, naming the noun or verb that semantically does not fit two other items.	104
	Category fluency (Luteijn and Barelds, 2004)	Producing words of a given category (animals; professions) within 60 sec.	464, 394^a^
Grammar	Sentence completion (Satoer et al., 2019)	Sentence completion with a constituent.	105
	Action naming in sentence context (Rofes, 2012)	Retrieving and inflecting verbs depicted on black-and-white drawings with the introduction *The (wo)man*…	143
Reading	Reading (Van Ierschot, 2018)	Reading sentences aloud.	51
Writing	Writing (Van Ierschot, 2018)	Writing sentences to dictation.	51

*^a^The first number concerns the subtest Animals; the second number concerns the subtest Professions.*

### Electroencephalography Resting-State Registration

Electroencephalography was registered using 44-channel Schwarzer amplifiers (Natus Europe GmbH, Munich). Twenty-one scalp electrodes were applied according to the International 10–20 System, with or without a cap according to local protocol. Additional polygraphic channels were applied for artefact detection: electrooculography (EOG; two diagonally placed electrodes for eye movements), electrocardiography (ECG; chest electrode), and respiration (abdominal movements). EEG was recorded with electrode impedances ≤ 5 kΩ, a sampling frequency of 500 Hz, and with Cz as reference electrode.

At least 5 min of eyes-closed, resting-state EEG were recorded for every participant. If there were many artefacts, the duration was prolonged to 10 min. Participants were instructed to refrain from moving and to stay alert. Alertness was continuously monitored and acoustic stimuli were given when signs of drowsiness appeared. For (glioma) patients with suspected epileptiform activity, the resting-state registration was followed by the standard clinical EEG registration of at least 30 min (these data were not taken into account for our analyses).

### Analysis of Slow-Wave Activity

Evaluation of slow-wave activity was performed visually,^2^ the gold standard in clinical evaluation, and quantitatively for investigating a relation to language performance.

Visual EEG analysis was performed in BrainRT software (v.2.0; Rumst, Belgium, 2013) by one of the authors (PJC), a board-certified clinical neurophysiologist, who was blinded to the participants’ medical situation (brain tumour or healthy). The presence and characteristics of activity in the delta (0.5–4 Hz), theta (4–8 Hz), and alpha (8–12 Hz) frequency bands were determined with resting-state EEG data reformatted to display standard antero-posterior bipolar montage and common average montages. Subsequently, slow-wave activity was classified according to the Mayo Classification System (Mayo Clinic, 1998), complemented by guidelines of Lüders and Noachtar (2000). From this, four categories were created: normal, mild, moderate, and severe slow-wave activity. See Table 2 for their definitions. As pathological slow-wave activity is generally pronounced over a region of brain injury (De Jongh et al., 2003), the categories of visual EEG analysis are presented in relation to quantitatively analysed slow-wave activity over the affected area as described below.

**TABLE 2:** Classification of slow-wave activity in EEG by visual analysis.

Degrees of slow-wave activity	Category derived from the Mayo Classification System	Interpretation^a^
Normal		No abnormal slow-wave activity
Mild	Dysrhythmia grade I Dysrhythmia grade II	Intermittent non-specific theta or delta slowing < 50% of the recording: focal, bilateral, or diffuse with amplitudes < 60 μV
Moderate	Dysrhythmia grade III	Intermittent non-specific theta or delta slowing >50% of the recording: focal, bilateral, or diffuse with amplitudes >60 μV^b^
	Delta grade I	Persistent polymorphic (irregular) delta slowing: focal, bilateral, or diffuse with amplitudes < 30 μV
Severe	Delta grade II Delta grade III	Persistent polymorphic (irregular) delta slowing: focal, bilateral, or diffuse with amplitudes > 30 μV

*Persistent asymmetry in amplitude or frequency of > 50% between the hemispheres was also scored, but not included in the analysis.*

*^a^Complemented by guidelines of Lüders and Noachtar (2000).*

*^b^Dysrhythmia grade III can also refer to specific epileptiform activity (spikes, sharp waves, and spike-waves as well as recorded ictal patterns). Even though this symptom was scored during visual interpretation of EEG, it was not included for data analysis.*

Quantitative EEG analysis was performed in BrainVision Analyzer 2.0 (Brain Products GmbH, 2013). Artefact-free EEG (e.g., blinks/eye movements, muscle contraction artefacts) was selected by visual inspection (IB) and was segmented into 20, 2-s epochs for each participant. Raw EEG data were re-referenced to a new averaged reference consisting of 16 scalp electrodes: F3; F4; F7; F8; T3; T4; T5; T6; C3; C4; P3; P4; O1; O2; Fz; and Pz [excluding: Cz (reference electrode); Fp1 and Fp2 (to minimise excess muscle activity and eye-movement artefacts); A1 and A2 (do not register brain activity)]. Subsequently, the data were filtered (band-pass: 0.27–30 Hz, slope 24 dB/Oct; notch: 50 Hz, slope 24 dB/Oct). This type of filtering leaves a low absolute power of higher frequency bands within the filtered signal. For every patient, spectral analysis was performed on the 20 epochs of 1,000 points by a Fast Fourier Transform (FFT) for all 16 electrodes. After averaging the 20 FFTs, absolute power in the delta (0.5–4 Hz) and theta range (4–8 Hz) was normalised by dividing both by the absolute power in the total spectrum (0.5–70 Hz). Although the absolute power in the frequency range above 30 Hz will be very low, excluding the power in the assessment of the relative power could overestimate the observed relative power in the lower frequency bands. To correct for this, it was decided to use the broad band spectrum. Hence, quantitative analysis of slow-wave activity resulted in relative power values in the delta and theta band separately.

**FIGURE 1:**
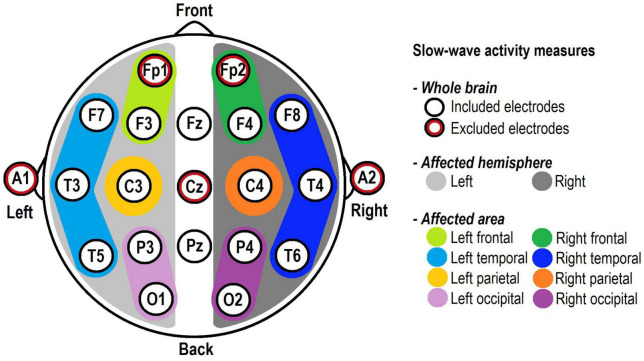
Electrode positions according to the International 10–20 System and their involvement in the slow-wave activity measures. The five red-circled electrodes were excluded from all analyses. “Whole brain” included the 16 remaining electrodes, whereas “Affected hemisphere” and “Affected area” included the electrodes corresponding to each patient’s individual tumour location.

In order to examine slow-wave activity at different spatial levels over the scalp, three measures were created. First, a whole-brain measure was computed, consisting of the mean activity of the 16 remaining scalp electrodes. Subsequently, two “tumour-specific” measures were created, taking into account the tumour localisation of individual patients: (De Robles et al., 2015) “Affected hemisphere,” consisting of the mean activity of seven electrodes on the side of the tumour (F3, F7, T3, T5, C3, P3, and O1 for left-sided tumours and F4, F8, T4, T6, C4, P4, and O2 for right-sided tumours); and (Davie et al., 2009) “Affected area,” consisting of the mean activity of electrodes over the region of the tumour, which was classified by a neuro-radiologist. This corresponded to one or two of the following regions (based on EEG regions as used in Gallego-Jutglà et al., 2015): left frontal (F3); right frontal (F4); left temporal (mean of F7, T3, and T5); right temporal (mean of F8, T4, and T6); left parietal (C3); right parietal (C4); left occipital (mean of P3 and O3); and right occipital (mean of P4 and O2; see Figure 1).

### Statistical Analysis

Analyses were performed using IBM SPSS Statistics software (IBM, 2016). For language performance, patients’ domain z-scores were compared to pre-existing normative data from a healthy population (*M* = 0) by one-sample Wilcoxon signed-rank tests. With regard to slow-wave activity, i.e., delta and theta activity over the whole brain, the patient groups were compared to the control groups by using Mann-Whitney *U* tests. As the two tumour-specific variables could only be calculated for patients, these variables were compared to the median of the whole-brain measure of the control groups, by using one-sample Wilcoxon signed rank tests. These tests were performed after confirmation that in the control groups there were no differences between the hemispheres (*N* = 15, delta: *Z* = −1.307, *p* = 0.191, theta: *Z* = −1.367, *p* = 0.172; *N* = 9, delta: *Z* = −0.771, *p* = 0.441, theta: *Z* = −0.475, *p* = 0.635). Only the slow-wave activity measures for which patients showed significantly more delta/theta activity than healthy individuals were used in further analyses concerning language functioning. Language scores were divided into categories “impaired” (z-score ≤ −1.5 on one or more language domains) or “unimpaired” (z-score ≥ −1.5): slow-wave activity of patients with language impairment was compared to slow-wave activity of patients without language impairment with Mann-Whitney *U* tests. Also, the language domain scores were analysed in relation to slow-wave activity with Kendall’s tau-b correlation coefficients.^3^ A significance level of 0.05 was used in all tests.

## Results

### Participants

Demographic and clinical characteristics of the 15 glioma patients (matched to 15 healthy individuals) and the 10 meningioma patients (matched to nine healthy individuals) can be found in Table 3. At T2, there was a drop-out of two glioma patients, due to severe side effects of adjuvant treatment, and two meningioma patients, because they were no longer motivated to participate in the study.

**TABLE 3:** Demographic and clinical characteristics of the participants: number of participants (and percentage) or mean (and range).

	Glioma patients (*N* = 15)	Control group matched to glioma patients (*N* = 15)	Meningioma patients (*N* = 10)	Control group matched to meningioma patients (*N* = 9)
Gender–female	5 (33%)	6 (40%)	6 (60%)	4 (44%)
Mean age in years (range)	42.0 (22–60)	42.3 (20–59)	58.6 (50–69)	53.8 (46–59)
Mean education level^a^ (range)	5.3 (4–7)	5.4 (4–7)	5.4 (3–7)	5.2 (4–7)
**Handedness^b^**				
Right	10 (67%)	12 (80%)	9 (90%)	8 (89%)
Left	4 (27%)	3 (20%)	0	1 (11%)
Ambidextrous	1 (7%)	0	1 (10%)	0
Mean “diagnosis to surgery” time in months (range)	20.8 (1.1–167.3)	NA	8.0 (2.1–49.7)	NA
**Tumour histology and grade^c^**				
Diffuse astrocytoma, grade II	5 (33%)	NA	NA	NA
Oligodendroglioma, grade II	10 (67%)	NA	NA	NA
Meningioma, grade I	NA	NA	10 (100%)	NA
**Tumour localisation, hemisphere**				
Left	13 (87%)	NA	10 (100%)	NA
Right^d^	2 (13%)	NA	0	NA
**Tumour localisation, lobes**				
Frontal	5 (33%)	NA	6 (60%)	NA
Fronto-temporal	1 (7%)	NA	0	NA
Fronto-parietal	1 (7%)	NA	0	NA
Temporal	1 (7%)	NA	0	NA
Temporo-insular	3 (20%)	NA	0	NA
Parietal	3 (20%)	NA	3 (30%)	NA
Parieto-temporal	1 (7%)	NA	0	NA
Parieto-occipital	0	NA	1 (10%)	NA
**Extent of resection^e^**				
Partial: 20–89%	7 (47%)	NA	1 (10%)	NA
Subtotal: 90–99%	6 (40%)	NA	1 (10%)	NA
Total: 100%	2 (13%)	NA	8 (80%)	NA
Use of anti-epileptic drugs at T1	13 (87%)	NA	6 (60%)	NA
Use of anti-epileptic drugs at T2	12/13 (92%)	NA	7/8 (88%)	NA
Postoperative glioma treatment (chemo/ radiotherapy ongoing or completed at T2)	10/13 (77%)	NA	NA	NA

*NA, not applicable; T1, before surgery; T2, 1 year after surgery.*

*Some variables add up to 101% due to rounding.*

*^a^Education level was classified according to seven categories (1 = incomplete primary education; 2 = complete primary education; 3 = primary education and <2 years of low-level secondary education; 4 = complete low-level secondary education; 5 = complete average-level secondary education; 6 = complete high-level secondary education; 7 = complete university education) (Verhage, 1964).*

*^b^Handedness was assessed by the Edinburgh Handedness Inventory (Oldfield, 1971).*

*^c^Based on the 2016 WHO (World Health Organisation) classification (Louis et al., 2016).*

*^d^The two right-hemispheric glioma patients had bilateral language activation in fMRI.*

*^e^Extent of resection was determined by a neuroradiologist, based on pre- and postoperative tumour volumes.*

### Language Performance

#### Language Performance in Glioma Patients: Pre- and Postoperatively

##### T1 and T2

Pre- and postoperative language domain z-scores are presented in Table 4. At T1 glioma patients scored significantly lower than the healthy population on the domains of Word retrieval, Phonology, Semantics, and Writing. A trend was found for Grammar (*p* = 0.063). On Reading, patients scored higher than the healthy population, as all but one patient made no errors on this test. At T2, patients had recovered on Word retrieval, whereas Grammar had become impaired. At the individual level, 9 patients (60%) had a language impairment at T1 and 10 patients (77%) had a language impairment at T2.

**TABLE 4:** Language domain z-scores of glioma patients at T1 and T2, including comparisons to normative data from a healthy population.

Language domain	T1: Language z-scores				Comparisons to a healthy population		T2: Language z-scores				Comparisons to a healthy population	
	*N*	*Mdn*	*Min*	*Max*	*Z*	*p*	*N*	*Mdn*	*Min*	*Max*	*Z*	*p*
Word Retrieval	15	−1.64	−10.71	0.83	−1.99	**0.023**	13	0.02	−7.18	0.88	−1.23	0.110
Phonology	15	−0.20	−5.28	0.95	−2.27	**0.012**	13	−0.84	−5.62	0.85	−2.20	**0.014**
Semantics	15	−0.60	−5.14	1.11	−1.87	**0.031**	13	−0.70	−3.99	0.91	−1.85	**0.032**
Grammar	15	−0.25	−8.33	0.74	−1.53	0.063	13	−0.59	−5.38	0.86	−2.41	**0.008**
Reading	14	0.28	−5.28	0.28	2.67	**0.004**	13	0.28	−2.50	0.28	−0.69	0.247
Writing	13	−0.79	−6.12	0.55	−2.16	**0.016**	13	−0.79	−7.45	0.55	−2.78	**0.003**

*N, sample size; Mdn, median; Min, minimum value; Max, maximum value; Z, standardised test statistic of the Wilcoxon signed rank tests; p, p-value (one-sided). Significant effects (p < 0.05) are presented in bold font.*

#### Language Performance in Meningioma Patients: Pre- and Postoperatively

##### T1 and T2

Pre- and postoperative language domain z-scores are presented in Table 5. At T1, meningioma patients scored significantly lower than the healthy population on domains of Word retrieval, Grammar, and Writing. On Reading, patients scored higher than the healthy population, as all but one patient made no errors on this test. At T2, performance on all tests had recovered apart from Writing. At the individual level, four patients (40%) had a language impairment at T1 and five patients (63%) had a language impairment at T2.

**TABLE 5:** Language domain z-scores of meningioma patients at T1 and T2, including comparisons to normative data from a healthy population.

Language domain	T1: Language z-scores				Comparisons to a healthy population		T2: Language z-scores				Comparisons to a healthy population	
	*N*	*Mdn*	*Min*	*Max*	*Z*	*p*	*N*	*Mdn*	*Min*	*Max*	*Z*	*p*
Word Retrieval	10	−0.58	−1.64	0.42	−1.89	**0.030**	8	−0.58	−8.44	0.88	−1.40	0.081
Phonology	10	−0.12	−1.34	1.16	−0.97	0.167	8	−0.11	−1.61	1.31	−0.14	0.445
Semantics	10	−0.48	−1.58	1.03	−1.27	0.102	8	0.38	−1.34	1.43	1.26	0.104
Grammar	10	−0.51	−1.29	−0.35	−2.81	**0.003**	8	−0.16	−3.77	0.43	−1.12	0.132
Reading	9	0.28	−2.50	0.28	1.73	**0.042**	8	0.28	−2.50	0.28	1.51	0.066
Writing	9	−0.79	−6.12	0.55	−1.98	**0.024**	8	−1.45	−6.12	0.55	−1.69	**0.046**

*N, sample size; Mdn, median; Min, minimum value; Max, maximum value; Z, standardised test statistic of the one-sample Wilcoxon signed rank tests; p, p-value (one-sided).*

*Significant effects (p < 0.05) are presented in bold font.*

#### Slow-Wave Activity in Glioma Patients: Pre- and Postoperatively

##### T1

**FIGURE 2:**
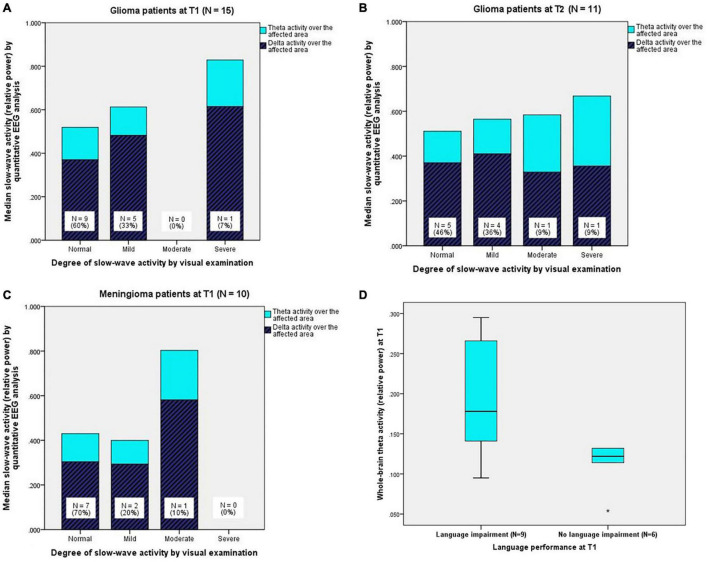
**(A)** (*Upper left*) Quantitatively analysed slow-wave activity (relative delta and theta power) over the affected area for every degree of slow-wave activity from visual EEG examination in glioma patients at T1 and **(B)** (*upper right*) at T2. **(C)** *(Below left)* Quantitatively analysed slow-wave activity (relative delta and theta power) over the affected area for every degree of slow-wave activity from visual EEG examination in meningioma patients at T1 (N.B. Meningioma patients did not undergo EEG registration at T2). **(D)** *(Below right)* Boxplots of theta activity over the whole brain for glioma patients with language impairment (*N* = 9) and glioma patients without language impairment (*N* = 6) at T1. The boxes contain 50% of the data (interquartile range), including the median (black horizontal line), and the vertically extending lines (whiskers) present the upper and lower quartiles of the data. “*” indicates an outlier > three times the interquartile range.

Upon visual EEG examination, nine patients (60%) had no abnormal slow-wave activity (“normal” in Table 2), five patients (33%) had a mild degree of slow-wave activity, and one patient (7%) had a severe degree of slow-wave activity. For every degree, Figure 2A shows the corresponding quantitative EEG results, namely, the median ratios of delta and theta activity over the affected area.

Quantitative EEG analysis revealed that glioma patients had significantly more delta activity over the affected area (*Mdn_*G*_^4^* = 0.450) compared to the whole-brain measure of the control group (*Mdn*_*C*_ = 0.324), *Z* = 2.158, *p* = 0.016, *r* = 0.56. No differences between the groups were found for delta activity over the whole brain and the affected hemisphere. Therefore, delta activity over the whole brain and the affected hemisphere at T1 were not analysed in relation to language performance.

In the theta band, glioma patients showed significantly more activity over the whole brain (*Mdn*_*G*_ = 0.132, *Mdn*_*C*_ = 0.110, *U* = 68.0, *p* = 0.034, *r* = 0.34), the affected hemisphere (*Mdn*_*G*_ = 0.135, *Mdn_*C*_* = 0.110, *Z* = 2.784, *p* = 0.003, *r* = 0.72), and the affected area (*Mdn*_*G*_ = 0.149, *Mdn*_*C*_ = 0.110, *Z* = 2.897, *p* = 0.002, *r* = 0.75) than healthy individuals. See Supplementary Appendix A1.

##### T2

One year after surgery, 11 glioma patients underwent a second EEG registration. Visual EEG examination showed that five patients (46%) had no abnormal slow-wave activity, four patients (36%) had a mild degree of slow-wave activity, one patient (9%) had a moderate degree of slow-wave activity, and one patient (9%) had a severe degree of slow-wave activity. For every degree, Figure 2B shows the corresponding, quantitatively analysed median ratios of delta and theta activity over the affected area.

Quantitative EEG analysis revealed that glioma patients had significantly more delta activity over the affected area (*Mdn*_*G*_ = 0.370) at T2 compared to the whole-brain measure of the control group (*Mdn*_*C*_ = 0.324), *Z* = 1.778, *p* = 0.038, *r* = 0.54). No differences between the groups were found for delta activity over the whole brain and the affected hemisphere.

In the theta band, glioma patients showed significantly more activity over the whole brain (*Mdn*_*G*_ = 0.139, *Mdn*_*C*_ = 0.110, *U* = 47.0, *p* = 0.035, *r* = 0.36), the affected hemisphere (*Mdn_*G*_* = 0.149, *Mdn*_*C*_ = 0.110, *Z* = 2.223, *p* = 0.013, *r* = 0.67), and the affected area (*Mdn*_*G*_ = 0.160, *Mdn*_*C*_ = 0.110, *Z* = 2.667, *p* = 0.004, *r* = 0.80) than healthy individuals. See Supplementary Appendix A1.

#### Slow-Wave Activity in Meningioma Patients: Pre- and Postoperatively

##### T1

Visual EEG examination revealed that seven patients (70%) had no abnormal slow-wave activity, two patients (20%) had a mild degree of slow-wave activity, and one patient (10%) had a moderate degree of slow-wave activity. For every degree, Figure 2C shows the corresponding, quantitatively analysed median ratios of delta and theta activity over the affected area.

Quantitative EEG analysis demonstrated that delta and theta activity over the whole brain, the affected hemisphere, and the affected area in meningioma patients did not differ from the whole-brain measure of the control group (Supplementary Appendix A2). Therefore, slow-wave activity in meningioma patients was not analysed in relation to language performance.

#### Preoperative Slow-Wave Activity and Preoperative Language Performance

Only those slow-wave activity measures for which patients had significantly more delta/theta activity than healthy individuals were used to examine whether increased preoperative slow-wave activity was related to preoperative language functioning.

#### Glioma Patients

##### Impaired vs. Unimpaired Preoperative Language Performance

No difference in delta activity over the affected area was found between glioma patients with and without language impairment at T1.

In the theta band, glioma patients with language impairment (*N* = 9) had significantly more activity over the whole brain (*Mdn*_*Imp*_ = 0.178, *Mdn_*Unimp*_* = 0.122, *U* = 8.5, *p* = 0.029, *r* = 0.56; see Figure 2D), the affected hemisphere (*Mdn*_*Imp*_ = 0.185, *Mdn_*Unimp*_* = 0.125, *U* = 10.0, *p* = 0.045, *r* = 0.52), and the affected area (*Mdn*_*Imp*_ = 0.214, *Mdn_*Unimp*_* = 0.131, *U* = 10.0, *p* = 0.045, *r* = 0.52) compared to glioma patients without language impairment (*N* = 6) at T1. See Supplementary Appendix A3.

##### Language Domains

No significant correlations were found between delta activity over the affected area and the language domain scores at T1.

**FIGURE 3:**
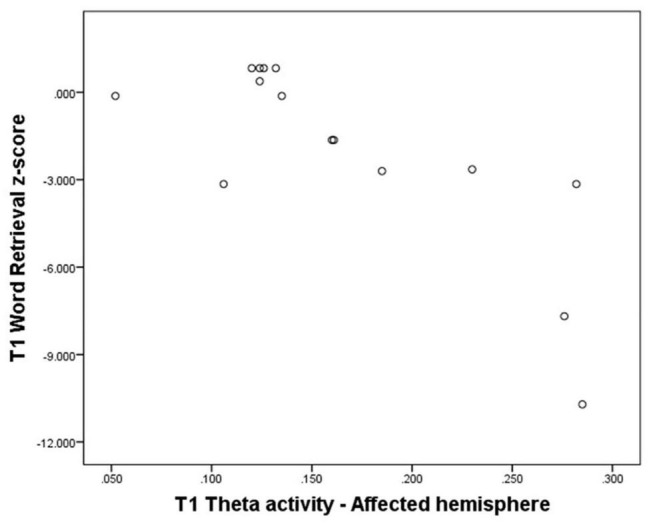
Theta activity over the affected hemisphere at T1 - Word retrieval domain z-score at T1 (*N* = 15).

Theta activity over the whole brain, the affected hemisphere (see for illustration scatterplot in Figure 3), and the affected area all showed a significant negative correlation with Word Retrieval scores (*N* = 15, *T* = −0.57, *p* = 0.004; *N* = 15, *T* = −0.57, *p* = 0.004; *N* = 15, *T* = −0.51; *p* = 0.010). Also, theta activity over the affected hemisphere showed a significant negative correlation with Grammar scores (*N* = 15, *T* = −0.53, *p* = 0.006), significant negative correlations were also found for the whole brain (*N* = 15, *T* = −0.47, *p* = 0.017) and the affected area (*N* = 15, *T* = −0.44; *p* = 0.025). See Supplementary Appendix A3.

#### Meningioma Patients

Slow-wave activity in meningioma patients was not analysed in relation to language performance because patients did not have more delta and theta activity than the control group (Supplementary Appendix A2).

#### Preoperative Slow-Wave Activity and Postoperative Language Outcome

Only those slow-wave activity measures for which patients had significantly more delta/theta activity than healthy individuals were used to examine whether increased preoperative slow-wave activity is related to 1 year postoperative language outcome.

#### Glioma Patients

##### Impaired vs. Unimpaired Postoperative Language Performance

No differences in slow-wave activity at T1 were found between glioma patients with and without language impairment at T2. See Supplementary Appendix A4.

##### Language Domains

No significant correlations were found between delta activity over the affected area at T1 and language scores at T2.

In the theta band, negative correlations were found between activity over the whole brain (*N* = 15, *T* = −0.51, *p* = 0.019), the affected hemisphere (*N* = 15, *T* = −0.53, *p* = 0.013), and the affected area (*N* = 15, *T* = −0.48; *p* = 0.026) and Word Retrieval scores at T2. See Supplementary Appendix A4.

## Discussion

The results indicate that low-grade glioma patients have increased slow-wave activity compared to healthy individuals, before and 1 year after surgery. Increased preoperative slow-wave activity is associated with the presence of language impairment and with poor language performance in specific language domains. Slow-wave activity before surgery is also related to language outcome (Word Retrieval) after surgery. Furthermore, meningioma patients have no increased slow-wave activity compared to healthy individuals, but they do show language impairments, before and after surgery. Below, we will address our formulated research questions in detail.

*(1) Do low-grade glioma and meningioma patients suffer from language impairments?*

### Language Performance in Glioma Patients: Pre- and Postoperatively

As expected, the low-grade glioma patients in this study have impaired language functioning at the group level before surgery. Compared to a healthy population, patients have poorer performance in almost all domains: word retrieval, phonology, semantics, and grammar. The patient group’s reading performance is better than that of a healthy population. This can be explained by the test being relatively easy as the normative data hardly include any errors and all but one patient reached the maximum score. With regard to the different language domains, impaired group-level performance in word retrieval, phonology, and semantics has previously been found in preoperative glioma patients, whereas impaired writing and comprehension of auditory input are less common (Satoer et al., 2014). Writing abilities have received little attention in patients undergoing awake glioma surgery (Van Ierschot et al., 2018), and when these abilities are evaluated preoperatively, they usually appear to be unimpaired (Santini et al., 2012; Satoer et al., 2014). The currently used writing test of Van Ierschot (2018) is presumably more sensitive to detect writing impairments than previously used writing subtests from aphasia batteries. In the year after surgery, performance on object naming recovered to a normative level, in line with earlier studies (Santini et al., 2012; Satoer et al., 2014), whereas the grammar domain, including a sentence completion test, deteriorates. This parallels a spontaneous speech analysis in which the parameter “number of incomplete sentences” significantly decreased compared to healthy controls (Satoer et al., 2018).

### Language Performance in Meningioma Patients: Pre- and Postoperatively

Before surgery, patients with a grade I meningioma have impairments in several language domains. Word-retrieval impairments have previously been found in meningioma patients preoperatively (Di Cristofori et al., 2018), which is in line with our results. Grammatical abilities and writing skills, however, have not been investigated in meningioma patients before. One year after surgery, at the group level, patients’ language performance in all investigated domains had recovered and was at the level of a healthy population, except for the writing abilities. This finding is in accordance with two other studies reporting on improvement in language skills (range: 2–12 months after surgery; Liouta et al., 2016; Di Cristofori et al., 2018). Hence, meningioma resection in the vicinity of eloquent brain areas does not seem to cause language deterioration at the group level. However, our data also show that testing of reading and writing is a useful addition to the tests already used at the spoken level.

*(2) Do low-grade glioma and meningioma patients have more slow-wave activity than healthy individuals in their EEG preoperatively and 1 year postoperatively?*

### Slow-Wave Activity in Glioma Patients: Pre- and Postoperatively

Glioma patients show more delta activity over the affected area, and more theta activity over the whole brain, the affected hemisphere, and the affected area before surgery. Pathologically increased slow-wave activity has already been shown to occur preoperatively in heterogeneous patient groups with different tumour types and tumour grades (Baayen et al., 2003; De Jongh et al., 2003; Oshino et al., 2007) and is now confirmed specifically for low-grade glioma patients. Nevertheless, visual EEG Examination does confirm much individual variation in the level of slow-wave activity.

Explanations for the observed slow-wave activity patterns are discussed for the delta and theta band separately. Presence of increased delta activity only over the affected area is in line with previous studies (Baayen et al., 2003; De Jongh et al., 2003; Oshino et al., 2007). Moreover, the findings, supported by Fernández-Bouzas et al. (1999), show that sources of delta activity are localised in the tumour margins. Literature on acute brain injury reports that increased localised delta activity is presumed to originate from injured tissue around the lesion, in particular, subcortical white-matter injury. Subcortical lesions appear to induce delta waves that are generated by pyramidal neurons in cortical areas overlying the lesion (partial cortical deafferentation; Ball et al., 1977; Gloor et al., 1977). Furthermore, after severe traumatic brain injury, the extent of delta activity is related to the magnitude of white-matter injury (Thatcher, 2006). Gliomas contain more glial cells than cortical grey matter and hence predominantly affect subcortical white matter (Larjavaara et al., 2007). This finding suggests that in glioma patients, white-matter injury contributes to emerging delta activity.

Increased theta activity is observed at all three spatial levels in our glioma-patient group. This findings agrees with those of Oshino et al. (2007), which assert that enhanced theta activity is present more diffusely than delta activity in preoperative brain-tumour patients. An explanation for the difference in spatial distribution between delta and theta activity is that the latter may be a result of the extent to which neural plasticity, that is, functional reorganisation of the brain, has succeeded (Duffau, 2014). In this line of reasoning, neural reorganisation was less successful in patients with residual postoperative language impairments compared to patients without postoperative language deficits. Increased theta activity may, thus, be a marker of this change in brain activity.

This current study is the first demonstrating that increased slow-wave activity is still present 1 year after glioma surgery. Moreover, the slow-wave activity patterns are identical to those before surgery (more delta activity over the affected area, and more theta activity over the whole brain, the affected hemisphere, and the affected area). De Jongh et al. (2003) reported similar patterns of delta activity preoperatively compared to 8 months postoperatively in a subgroup of patients with different types of low-grade brain tumours. Together, the findings suggest that in the long-term, brain tissue around the surgical cavity and/or residual tumour remains injured or dysfunctional. Alternatively, increased slow-wave activity 1 year after surgery may be associated with adjuvant treatment, which the majority of glioma patients in our study received around the time of testing (Nokia et al., 2012).

### Slow-Wave Activity in Meningioma Patients: Pre- and Postoperatively

Unlike glioma patients, meningioma patients have no preoperative abnormal slow-wave activity at the group level, as was seen in the study conducted by Oshino et al. (2007). In their study, meningiomas were associated with less delta activity and less consistent delta source locations than intra-axial tumours (gliomas and metastatic tumours), while correcting for tumour size and surrounding oedema. The difference between tumour types is presumably due to the fact that meningiomas are not infiltrative but only cause mass effect on surrounding brain tissue, especially on subcortical tissue, and that white matter pathways tend to be displaced but intact (Campanella et al., 2014). Therefore, meningiomas cause minimal or no white-matter injury, hence, only minimal or no delta activity (Gloor et al., 1977).

*(3) Is increased slow wave activity related to preoperative language functioning and 1 year postoperative language outcome?*

### Preoperative Slow-Wave Activity and Preoperative Language Performance

Glioma patients with language impairment have similar delta activity, but more theta activity over the whole brain, the affected hemisphere and the affected area, compared to glioma patients without language impairment. This finding suggests that impaired and unimpaired language functioning can be further supported on the basis of theta activity level, which is a novel finding in brain tumour patients. Similar results have been reported for other patient groups. In post-stroke aphasic individuals can be discriminated from post-stroke non-aphasic individuals by increased left-hemispheric delta activity, maximally over perisylvian areas (Chapman et al., 1989). The localisation of enhanced slow-wave activity is in agreement with the current results, as the affected hemisphere is generally the left hemisphere and the affected area commonly lies around the Sylvian fissure. However, the frequency band (delta instead of theta) that Chapman et al. (1989) report is not in line with our findings. This result may be explained by the different types of brain injury (acute vs. non-acute) and by the fact that Chapman et al. (1989) did not include theta activity. Also, individuals with non-acute, neurodegenerative language impairments (e.g., primary progressive aphasia) can be differentiated from healthy, age-matched individuals by slow-wave activity patterns, more specifically, by increased delta activity and increased theta activity over left-hemispheric language-related areas (Kielar et al., 2019; Shah-Basak et al., 2019). Overall, previous studies and the current study point in the same direction: increased slow-wave activity, at least theta activity, is related to language impairments.

When considering different language domains, increased theta activity is associated with poorer word retrieval and grammatical abilities. The relation between increased theta activity and poor word retrieval, as assessed with an object-naming test, has previously been found in patients with Alzheimer’s disease (Kim et al., 2012), although not consistently (Helkala et al., 1991; Duffy et al., 1995; Van der Hiele et al., 2007) and in patients with traumatic brain injury (Thatcher et al., 1998). No significant correlations have been reported for delta activity, similar to the current study. The relation between increased theta activity and affected grammatical abilities, as assessed with action naming in sentence context and sentence completion, has not been investigated before. Previous studies did not include tests on grammar nor did they analyse grammar separately in relation to slow-wave activity (Shah-Basak et al., 2019). In general, grammatical performance is only occasionally tested in brain tumour patients, even though it contributes important information to perioperative language assessments, as it reflects communication at the sentence level (Ohlerth, 2019).

### Preoperative Slow-Wave Activity and Postoperative Language Outcome

Our study showed that slow-wave activity in the theta band in low-grade glioma patients is predictive of 1 year postoperative language functioning, particularly with word retrieval (as assessed with object naming). This finding is line with earlier findings on the prognostic value of preoperative slow-wave activity on functional outcome after brain-tumour surgery (Oshino et al., 2007).

Several studies in stroke patients have also shown that increased slow-wave activity is indicative of poorer language recovery 2–24 months post-onset. Evidence has been provided for delta activity (Hensel et al., 2004), theta activity (Tikofsky et al., 1960), and delta-theta activity combined (Jabbari et al., 1979; Szelies et al., 2002). The discrepancy with our results (i.e., theta activity only) may be due to the differences in pathophysiology and the severity of the language impairment it induced: an acute stroke causing language impairments (previous studies), vs. a slow-growing tumour and its surgical resection, which caused language impairment only in some of the patients (current study). The lower rate of language impairment in low-grade glioma patients can be explained by the fact that a slowly growing tumour allows for neuroplasticity processes and reorganisation of (language) functions. Moreover, a low-grade brain tumour is generally associated with less acute brain injury and less severe language impairment than a stroke in the same location (Anderson et al., 1990).

### Methodological Considerations

The current results should be interpreted with caution because the sample sizes are small and there is a high level of inter-individual variability in language scores and slow-wave activity. Even though all registrations were visually inspected and checked for their quality, the slow-wave activity results may have been influenced by the use of anti-epileptic drugs at T1 and T2 and adjuvant cancer treatment at T2 (Duncan, 1987; Nokia et al., 2012). Furthermore, EEG registration comes with a few challenges. For example, EEG has relatively low spatial resolution and is sensitive to conductivity errors, as volume conduction through the skull and other tissues can blur the signal. Therefore, it is not certain that the signal registered at the scalp originates from the brain area exactly underneath. In addition, by following the standard clinical procedure, EEG was registered with 21 scalp electrodes, which limits the spatial resolution. However, an advantage of EEG in this context is that many brain-tumour patients already undergo EEG registration as part of their diagnostic examination, due to suspected epileptic seizures. In those instances, evaluation of slow-wave activity does not require much extra time. Finally, we filtered the EEG data after selecting artefact free epochs instead of on the continuous recording. we are aware that introducing and timing of filtering in the analysis can give rise to different filtering effects. It can improve the signal-to-noise-ratio but on the other hand it can also give rise to distortion which can be of concern, especially in ERP studies or analysing seizure detection on EEG recordings.

## Conclusion

This study is the first prospective study to report on slow-wave brain activity in low-grade glioma and meningioma patients and its relation to language functioning. Patients with low-grade gliomas and meningiomas both suffer from language impairments, however, the brain tumour pathology has different effects on resting-state brain activity. Increased theta activity in glioma patients can be considered as a marker of language impairment, with prognostic value for language outcome after surgery. The search for predictors of language outcome in brain tumour patients remains important because new insights may aid (peri)surgical procedures and potentially help to prevent language decline.

## Data Availability Statement

The original contributions presented in the study are included in the article/Supplementary Material, further inquiries can be directed to the corresponding author/s.

## Ethics Statement

The studies involving human participants were reviewed and approved by the Medical Ethical Review Board of University Medical Centre Groningen and Medical Ethical Review Board of Erasmus Medical Center in Rotterdam, Netherlands. The patients/participants provided their written informed consent to participate in this study.

## Author Contributions

RB, IB, PC, and DS: conception and design study. NW, WV, DS, IB, PC, MW, AV, and MS: data collection. NW, IB, PC, MS, and DS: analyses and interpretation. NW, RB, IB, PC, and DS: drafting article. RB, IB, PC, and DS: project supervision. NW, IB, RB, PC, MS, WV, MW, AV, and DS: critical article revision. All authors contributed to the article and approved the submitted version.

## Conflict of Interest

The authors declare that the research was conducted in the absence of any commercial or financial relationships that could be construed as a potential conflict of interest.

## Publisher’s Note

All claims expressed in this article are solely those of the authors and do not necessarily represent those of their affiliated organizations, or those of the publisher, the editors and the reviewers. Any product that may be evaluated in this article, or claim that may be made by its manufacturer, is not guaranteed or endorsed by the publisher.

## Footnotes

1 As we expect no language impairments after surgery in the meningioma group due to brain compression by the tumour instead of infiltration, we did not perform EEG recordings at T2.

2 Visually selecting artefact free EEG epochs is the gold standard to approach automated EEG signal analysis (rather than post-processing to detect and remove using techniques such as independent component analysis or blind source separation). Such approaches are mostly needed when we analyse seizures recorded on EEG, which could be often contaminated by muscle contraction and movement artefacts. Our study population consisted of co-operative patients in whom it was possible to obtain artefact free EEG recordings.

3 The Kendall’s tau-b test was selected because it best handles the combination of small sample sizes and many ties (patients with identical language scores) in the data, which do not meet the assumptions for parametric testing.

4 *Mdn*_*G*_, *Median Gliomas*; *Mdn*_*C*_, *Median Controls.*
