# Supplementary material for: Distinct Slow-Wave Activity Patterns in Resting-State Electroencephalography and Their Relation to Language Functioning in Low-Grade Glioma and Meningioma Patients
Source: Front Hum Neurosci. 2022 Mar 24;16:748128. doi: 10.3389/fnhum.2022.748128 (PMC8986989; doi:10.3389/fnhum.2022.748128)
Supplement: Supplementary file 1 [file Data_Sheet_1.docx]

**Appendix A1 – Slow-wave activity in glioma patients**

**T1: before surgery**

*Whole-brain and tumour-specific measures of slow-wave activity in glioma patients compared to the whole-brain measure of the control group*

|  | **Glioma patients (*N* = 15)** | | |  | **Control group (*N* = 15)** | | |  |  | | |
| --- | --- | --- | --- | --- | --- | --- | --- | --- | --- | --- | --- |
|  | Whole brain | | |  | Whole brain | | |  | Comparisons^a^ | | |
|  | *Mdn* | *Min* | *Max* |  | *Mdn* | *Min* | *Max* |  | *U* | *p* | *r* |
| Delta | 0.370 | 0.150 | 0.534 |  | 0.324 | 0.212 | 0.590 |  | 103.0 | 0.357 | 0.07 |
| Theta | 0.132 | 0.054 | 0.295 |  | 0.110 | 0.084 | 0.183 |  | 68.0 | **0.034** | 0.34 |
|  | Affected hemisphere | | |  | Whole brain | | |  | Comparisons^b^ | | |
|  | *Mdn* | *Min* | *Max* |  | *Mdn* | *Min* | *Max* |  | *Z* | *p* | *r* |
| Delta | 0.408 | 0.157 | 0.548 |  | 0.324 | 0.212 | 0.590 |  | 1.420 | 0.078 | 0.37 |
| Theta | 0.135 | 0.052 | 0.285 |  | 0.110 | 0.084 | 0.183 |  | 2.784 | **0.003** | 0.72 |
|  | Affected area | | |  | Whole brain | | |  | Comparisons^b^ | | |
|  | *Mdn* | *Min* | *Max* |  | *Mdn* | *Min* | *Max* |  | *Z* | *p* | *r* |
| Delta | 0.450 | 0.215 | 0.615 |  | 0.324 | 0.212 | 0.590 |  | 2.158 | **0.016** | 0.56 |
| Theta | 0.149 | 0.055 | 0.322 |  | 0.110 | 0.084 | 0.183 |  | 2.897 | **0.002** | 0.75 |

*Note*. *Mdn* = median; *Min* = minimum value; *Max* = maximum value; *U* = test statistic of the Mann-Whitney U tests; *Z* = standardised test statistic of the one-sample Wilcoxon signed rank tests; *p* = p-value (one-sided); *r* = effect size ($r=Z/\sqrt{N}$). Significant effects (*p* < 0.05) are presented in bold font. ^a^ Comparisons with Mann-Whitney U tests. ^b^ Slow-wave activity over the affected hemisphere and the affected area in glioma patients was compared to the median of the whole-brain measure of the control group by one-sample Wilcoxon signed rank tests because the control group does not have affected hemispheres or areas.

**T2: 1 year after surgery**

*Whole-brain and tumour-specific measures of slow-wave activity at T2 in glioma patients compared to the whole-brain measure of the control group*

|  | **Glioma patients (*N* = 11)** | | |  | **Control group (*N* = 15)** | | |  |  | | |
| --- | --- | --- | --- | --- | --- | --- | --- | --- | --- | --- | --- |
|  | Whole brain | | |  | Whole brain | | |  | Comparisons^a^ | | |
|  | *Mdn* | *Min* | *Max* |  | *Mdn* | *Min* | *Max* |  | *U* | *p* | *r* |
| Delta | 0.315 | 0.219 | 0.482 |  | 0.324 | 0.212 | 0.590 |  | 77.0 | 0.400 | 0.06 |
| Theta | 0.139 | 0.069 | 0.329 |  | 0.110 | 0.084 | 0.183 |  | 47.0 | **0.035** | 0.36 |
|  | Affected hemisphere | | |  | Whole brain | | |  | Comparisons^b^ | | |
|  | *Mdn* | *Min* | *Max* |  | *Mdn* | *Min* | *Max* |  | *Z* | *p* | *r* |
| Delta | 0.336 | 0.216 | 0.466 |  | 0.324 | 0.212 | 0.590 |  | 0.979 | 0.164 | 0.30 |
| Theta | 0.149 | 0.068 | 0.324 |  | 0.110 | 0.084 | 0.183 |  | 2.223 | **0.013** | 0.67 |
|  | Affected area | | |  | Whole brain | | |  | Comparisons^b^ | | |
|  | *Mdn* | *Min* | *Max* |  | *Mdn* | *Min* | *Max* |  | *Z* | *p* | *r* |
| Delta | 0.370 | 0.218 | 0.638 |  | 0.324 | 0.212 | 0.590 |  | 1.778 | **0.038** | 0.54 |
| Theta | 0.160 | 0.089 | 0.367 |  | 0.110 | 0.084 | 0.183 |  | 2.667 | **0.004** | 0.80 |

*Note*. *Mdn* = median; *Min* = minimum value; *Max* = maximum value; *U* = test statistic of the Mann-Whitney U tests; *Z* = standardised test statistic of the one-sample Wilcoxon signed rank tests; *p* = p-value (one-sided); *r* = effect size ($r=Z/\sqrt{N}$). Significant effects (*p* < 0.05) are presented in bold font. ^a^ Comparisons with Mann-Whitney U tests.^b^ Slow-wave activity over the affected hemisphere and the affected area in glioma patients was compared to the median of the whole-brain measure of the control group by one-sample Wilcoxon signed rank tests because the control group does not have affected hemispheres or areas.

**Appendix A2 – Slow-wave activity in meningioma patients**

**T1: before surgery**

*Whole-brain and tumour-specific measures of slow-wave activity in meningioma patients compared to the whole-brain measure of the control group*

|  | **Meningioma patients (*N* = 10)** | | |  | **Control group (*N* = 9)** | | |  |  | | |
| --- | --- | --- | --- | --- | --- | --- | --- | --- | --- | --- | --- |
|  | Whole brain | | |  | Whole brain | | |  | Comparisons^a^ | | |
|  | *Mdn* | *Min* | *Max* |  | *Mdn* | *Min* | *Max* |  | *U* | *p* | *r* |
| Delta | 0.341 | 0.101 | 0.535 |  | 0.324 | 0.212 | 0.398 |  | 42.0 | 0.421 | 0.06 |
| Theta | 0.118 | 0.067 | 0.186 |  | 0.110 | 0.084 | 0.149 |  | 36.0 | 0.249 | 0.17 |
|  | Affected hemisphere | | |  | Whole brain | | |  | Comparisons^b^ | | |
|  | *Mdn* | *Min* | *Max* |  | *Mdn* | *Min* | *Max* |  | *Z* | *p* | *r* |
| Delta | 0.325 | 0.103 | 0.537 |  | 0.324 | 0.212 | 0.398 |  | -0.153 | 0.439 | 0.05 |
| Theta | 0.121 | 0.069 | 0.188 |  | 0.110 | 0.084 | 0.149 |  | 0.866 | 0.193 | 0.27 |
|  | Affected area | | |  | Whole brain | | |  | Comparisons^b^ | | |
|  | *Mdn* | *Min* | *Max* |  | *Mdn* | *Min* | *Max* |  | *Z* | *p* | *r* |
| Delta | 0.322 | 0.087 | 0.581 |  | 0.324 | 0.212 | 0.398 |  | -0.255 | 0.400 | 0.08 |
| Theta | 0.128 | 0.079 | 0.231 |  | 0.110 | 0.084 | 0.149 |  | 1.244 | 0.107 | 0.39 |

*Note*. *Mdn* = median; *Min* = minimum value; *Max* = maximum value; *U* = test statistic of the Mann-Whitney U tests; *Z* = standardised test statistic of the one-sample Wilcoxon signed rank tests; *p* = p-value (one-sided); *r* = effect size ($r=Z/\sqrt{N}$). ^a^ Comparisons with Mann-Whitney U tests.^b^ Slow-wave activity over the affected hemisphere and the affected area in glioma patients was compared to the median of the whole-brain measure of the control group by one-sample Wilcoxon signed rank tests because the control group does not have affected hemispheres or areas.

**Appendix A3 – Slow-wave activity at T1 and language performance at T1 in glioma patients**

**Impaired vs. unimpaired language performance**

*Slow-wave activity at T1: patients with language impairment compared to patients without language impairment at T1*

|  | Patients with  language impairment  (*N* = 9) | | |  | Patients without language impairment  (*N* = 6) | | |  | Comparisons | | |
| --- | --- | --- | --- | --- | --- | --- | --- | --- | --- | --- | --- |
|  | *Mdn* | *Min* | *Max* |  | *Mdn* | *Min* | *Max* |  | *U* | *p* | *r* |
| Delta-Affected area^a^ | 0.370 | 0.219 | 0.615 |  | 0.477 | 0.215 | 0.606 |  | 21.0 | 0.480 | 0.18 |
| Theta-Whole brain | 0.178 | 0.095 | 0.295 |  | 0.122 | 0.054 | 0.132 |  | 8.5 | **0.029** | 0.56 |
| Theta-Affected hemisphere | 0.185 | 0.106 | 0.285 |  | 0.125 | 0.052 | 0.135 |  | 10.0 | **0.045** | 0.52 |
| Theta-Affected area | 0.214 | 0.099 | 0.322 |  | 0.131 | 0.055 | 0.149 |  | 10.0 | **0.045** | 0.52 |

*Note*. *Mdn* = median; *Min* = minimum value; *Max* = maximum value; *U* = test statistic of the Mann-Whitney U tests; *p* = p-value (two-sided); *r* = effect size ($r=Z/\sqrt{N}$). Significant effects (*p* < 0.05) are presented in bold font.

^a^ In the delta band, activity over the whole brain and the affected hemisphere were not taken into account because glioma patients at T1 did not have more delta activity over those regions than the control group (see Appendix A1).

**Language domains**

*Correlation analyses between slow-wave activity at T1 and language domain scores at T1*

| Language scores  at T1 | Delta^a^  Affected area | | |  | Theta  Whole brain | | |  | Theta  Affected hemisphere | | |  | Theta  Affected area | | |
| --- | --- | --- | --- | --- | --- | --- | --- | --- | --- | --- | --- | --- | --- | --- | --- |
|  | *N* | *ᴛ* | *p* |  | *N* | *ᴛ* | *p* |  | *N* | *ᴛ* | *p* |  | *N* | *ᴛ* | *p* |
| Word Retrieval | 15 | 0.10 | 0.616 |  | 15 | -0.57 | **0.004** |  | 15 | -0.57 | **0.004** |  | 15 | -0.51 | **0.010** |
| Phonology | 15 | 0.14 | 0.458 |  | 15 | -0.14 | 0.457 |  | 15 | -0.12 | 0.552 |  | 15 | -0.10 | 0.620 |
| Semantics | 15 | 0.14 | 0.458 |  | 15 | -0.05 | 0.804 |  | 15 | -0.13 | 0.488 |  | 15 | -0.10 | 0.620 |
| Grammar | 15 | 0.14 | 0.487 |  | 15 | -0.47 | ***0.017*** |  | 15 | -0.53 | **0.006** |  | 15 | -0.44 | **0.025** |
| Reading | 14 | -0.09 | 0.710 |  | 14 | 0.18 | 0.456 |  | 14 | 0.26 | 0.264 |  | 14 | 0.26 | 0.264 |
| Writing | 13 | 0.05 | 0.845 |  | 13 | -0.27 | 0.239 |  | 13 | -0.33 | 0.150 |  | 13 | -0.21 | 0.360 |

*Note. N* = group size; *ᴛ* = Kendall’s tau-b correlation coefficient; *p* = p-value (two-sided). Significant effects (*p* < 0.05) are presented in bold font.

^a^ In the delta band, activity over the whole brain and the affected hemisphere were not taken into account because glioma patients at T1 did not have more delta activity over those regions than the control group (see Appendix D1).

N.B. The previous analyses were not performed for meningioma patients because they did not have more slow-wave activity than healthy individuals.

**Appendix A4 – Slow-wave activity at T1 and language outcome at T2 in glioma patients**

**Impaired vs. unimpaired language performance**

*Slow-wave activity at T1: patients with language impairment compared to patients without language impairment at T2*

|  | Patients with  language impairment  (*N* = 10) | | |  | Patients without language impairment  (*N* = 3) | | |  | Comparisons | | |
| --- | --- | --- | --- | --- | --- | --- | --- | --- | --- | --- | --- |
|  | *Mdn* | *Min* | *Max* |  | *Mdn* | *Min* | *Max* |  | *U* | *p* | *r* |
| Delta-Affected area^a^ | 0.413 | 0.219 | 0.615 |  | 0.526 | 0.215 | 0.606 |  | 12.0 | 0.612 | 0.14 |
| Theta-Whole brain | 0.160 | 0.054 | 0.295 |  | 0.123 | 0.114 | 0.132 |  | 7.5 | 0.204 | 0.35 |
| Theta-Affected hemisphere | 0.173 | 0.052 | 0.285 |  | 0.126 | 0.124 | 0.132 |  | 8.5 | 0.271 | 0.31 |
| Theta-Affected area | 0.200 | 0.055 | 0.322 |  | 0.131 | 0.131 | 0.137 |  | 9.0 | 0.310 | 0.28 |

*Note*. *Mdn* = median; *Min* = minimum value; *Max* = maximum value; *U* = test statistic of the Mann-Whitney U tests; *p* = p-value (two-sided); *r* = effect size ($r=Z/\sqrt{N}$).

^a^ In the delta band, activity over the whole brain and the affected hemisphere were not taken into account because glioma patients at T1 did not have more delta activity over those regions than the control group (see Appendix A1).

**Language domains**

*Correlation analyses between slow-wave activity at T1 and language outcome at T2*

| Language scores  at T2 | Delta  Affected area | | |  | Theta  Whole brain | | |  | Theta  Affected hemisphere | | |  | Theta  Affected area | | |
| --- | --- | --- | --- | --- | --- | --- | --- | --- | --- | --- | --- | --- | --- | --- | --- |
|  | *N* | *ᴛ* | *p* |  | *N* | *ᴛ* | *p* |  | *N* | *ᴛ* | *p* |  | *N* | *ᴛ* | *p* |
| Word Retrieval | 13 | 0.33 | 0.123 |  | 13 | -0.51 | **0.019** |  | 13 | -0.53 | **0.013** |  | 13 | -0.48 | **0.026** |
| Phonology | 13 | 0.26 | 0.222 |  | 13 | -0.16 | 0.462 |  | 13 | -0.09 | 0.669 |  | 13 | -0.07 | 0.760 |
| Semantics | 13 | 0.18 | 0.393 |  | 13 | -0.03 | 0.903 |  | 13 | -0.01 | 0.951 |  | 13 | 0.01 | 0.951 |
| Grammar | 13 | -0.04 | 0.854 |  | 13 | -0.01 | 0.951 |  | 13 | 0.00 | 1.000 |  | 13 | 0.05 | 0.806 |
| Reading | 13 | 0.18 | 0.464 |  | 13 | 0.04 | 0.883 |  | 13 | 0.09 | 0.714 |  | 13 | 0.04 | 0.883 |
| Writing | 13 | 0.13 | 0.567 |  | 13 | 0.07 | 0.750 |  | 13 | 0.03 | 0.899 |  | 13 | 0.12 | 0.611 |

*Note. N* = group size; *ᴛ* = Kendall’s tau-b correlation coefficient; *p* = p-value (two-sided). Significant effects (*p* < 0.05) are presented in bold font.

^a^ In the delta band, activity over the whole brain and the affected hemisphere were not taken into account because glioma patients at T1 did not have more delta activity over those regions than the control group (see Appendix D1).

N.B. The previous analyses were not performed for meningioma patients because they did not have more slow-wave activity than healthy individuals.
